# Structural basis of LaDR5, a novel agonistic anti-death receptor 5 (DR5) monoclonal antibody, to inhibit DR5/TRAIL complex formation

**DOI:** 10.1186/1471-2172-13-40

**Published:** 2012-07-12

**Authors:** Chunxia Qiao, Meiyun Hu, Leiming Guo, Ming Lv, Zhou Lin, Jing Geng, Xiaoling Lang, Xinying Li, Yan Li, Yuanfang Ma, Jiannan Feng, Beifen Shen

**Affiliations:** 1 Department of Immunology, Institute of Basic Medical Sciences, P.O. Box130(3), Taiping Road, Beijing, 100850, People’s Republic of China; 2 Laboratory of Cellular and Molecular Immunology, Institute of Immunology, Henan University, Kaifeng, 475001, People’s Republic of China

**Keywords:** TRAIL, Death receptor 5, Monoclonal antibody, Apoptosis, Breast cancer

## Abstract

**Background:**

As a member of the TNF superfamily, TRAIL could induce human tumor cell apoptosis through its cognate death receptors DR4 or DR5, which can induce formation of the death inducing signaling complex (DISC) and activation of the membrane proximal caspases (caspase-8 or caspase-10) and mitochondrial pathway. Some monoclonal antibodies against DR4 or DR5 have been reported to have anti-tumor activity.

**Results:**

In this study, we reported a novel mouse anti-human DR5 monoclonal antibody, named as LaDR5, which could compete with TRAIL to bind DR5 and induce the apoptosis of Jurkat cells in the absence of second cross-linking *in vitro*. Using computer-guided molecular modeling method, the 3-D structure of LaDR5 Fv fragment was constructed. According to the crystal structure of DR5, the 3-D complex structure of DR5 and LaDR5 was modeled using molecular docking method. Based on distance geometry method and intermolecular hydrogen bonding analysis, the key functional domain in DR5 was predicted and the DR5 mutants were designed. And then, three mutants of DR5 was expressed in prokaryotic system and purified by affinity chromatograph to determine the epitope of DR5 identified by LaDR5, which was consistent with the theoretical results of computer-aided analysis.

**Conclusions:**

Our results demonstrated the specific epitope located in DR5 that plays a crucial role in antibody binding and even antineoplastic bioactivity. Meanwhile, revealed structural features of DR5 may be important to design or screen novel drugs agonist DR5.

## Background

TRAIL (tumor necrosis factor (TNF)-related apoptosis-inducing ligand) is a member of the TNF superfamily with the ability to induce apoptosis of tumor cells. At least five receptors for TRAIL have been identified. DR4 (TRAIL-R1) [1] and DR5 (TRAIL-R2) [2-4] are apoptosis-inducing receptors. They contain an intracellular death domain each. Upon receptor activation, DR4 and DR5 recruit FAS associated protein with death domain (FADD) [5-7] and caspase-8 to form the death-inducing signaling complex (DISC), which activates caspase-8, subsequently leading to the activation of executioner caspases such as caspase-3 that induce apoptosis [5,8-10]. In addition, other signaling pathways leading to NF-kB activation and activation of the ERK, JNK, and p38 MAP kinase pathways are generated by a second complex that forms after the receptor-containing DISC [8]; DcR1 [2,3,11] and DcR2 [12,13] are nonsignaling decoy receptors, which can bind to TRAIL but cannot trigger apoptosis. DcR1 lacks cytoplasmic death domain entirely [14], whereas DcR2 has a nonfunctional truncated death domain [3,15]. DcR1 and DcR2 are unable to recruit FADD or signal apoptosis and inhibit apoptosis by sequestering TRAIL or by forming complexes with DR4 and DR5 to create heteromeric receptor complexes that are unable to activate signaling [16,17]; Another receptor, osteoprotegerin, is a soluble receptor that binds to TRAIL with a dissociation constant of 400 nM, which is the lowest affinity of the known TRAIL receptors [18]. It may have a more prominent role in bone and myeloid cell development than in regulating TRAIL-induced apoptosis.

A TRAIL monomer contains two anti-parallel beta-pleated sheets that form a beta-sandwich as a core scaffold and interacts with the adjacent subunits in a head-to-tail fashion to form a bell-shaped homo-trimmer. TRAIL and sDR5 form a tight 3:3 complex. Three sDR5 molecules in a curved shape bind to the three identical grooves between neighboring subunits of TRAIL, thereby inducing oligomerization of intracellular death domains. In complex TRAIL, residues 130–160 display the most remarkable structural changes. The conformation of residues 146–160 interact with the core scaffold and the residues 130–145 undergoes a drastic positional change. They penetrate into the receptor-binding site and interact with sDR5. This translocation is manifested by the strong electron densities observed for residues 130–135 of all the six TRAIL molecules in the asymmetric unit. Residues 131–135 penetrate into the central binding interface upon complex formation. The guanidino group of Arg^132^ makes a polar interaction with Tyr^50^ of sDR5. Asn^134^ and Thr^135^ interact with Glu^70^ and Asn^81^ of sDR5, respectively. The TRAIL mutant with residues 132–135 deletion showed a profound decrease in the binding affinity for sDR5 and cytotoxic activity *in vitro* using human hepatoma cells, suggesting those residues bear direct correlation with the activity of TRAIL [19].

TRAIL [20-22] and agonistic antibodies that recognize TRAIL receptors [23,24] preferentially kill tumor cells and produce potent anti-tumor activity in a variety of experimental models. It is therefore hoped that these agents may be useful to treat cancer [9,25-28]. Several clinical trials are ongoing with different TRAIL receptor agonists alone or in combination with other anti-cancer drugs. rTRAIL (soluble recombinant TRAIL), apoptosis-inducing anti-DR5/DR4 antibodies, or an agent that increases endogenous TRAIL expression are available strategies for cancer therapy [22,24,29]. Consistently, administration of TRAIL to mice bearing human tumors actively suppressed tumor progression [22] and improved survival of the animal. Furthermore, repeated intravenous injections of TRAIL in nonhuman primates did not cause detectable toxicity to normal tissues and organs, including liver tissues [21]. However, susceptibility of human normal hepatocytes to TRAIL was reported recently [30], therefore agonists against DR4 or DR5 (*e.g.* anti-DR5 antibody) by activating apoptosis signal are becoming dramatically meaningful as candidate drugs for cancer therapy.

Recently, preclinical studies of TRAIL Receptor agonists indicate that they may be efficacious in a wide range of tumor types, especially when combined with chemotherapeutic agents. Receptor agonists, including recombinant forms of TRAIL and monoclonal antibodies against DR4 or DR5, are currently being evaluated in Phase II clinical trials in several cancer indications, in which at least four human or humanized monoclonal antibodies that target DR5 have been undergoing evaluation in clinical trials: Lexatumumab (HGS-ETR2), Apomab, Conatumumab (AMG 655), Tigatuzumab (CS1008). Most of them could activates intracellular caspases and induce apoptosis in a wide spectrum of human cancer cell lines *in vitro* and have anti-tumor activity in a dose-dependent manner as a single agent in xenograft models. They also have synergistic or additive activity in combination with chemotherapeutic agents or radiation *in vitro* and/or *in vivo*. Results of a Phase I single-agent study showed that anti-DR5 antibodies were well tolerated, and some of them were studied about the safety and efficacy in a Phase II study of patients with cancer [31].

Many DR5 agonist antibodies require additional cross-linking to achieve optimal activity *in vitro*, such as Apomab and Tigatuzumab (CS1008) [32-34]. In xenograft models, this cross-linking function is likely provided by binding to FcγRs [23]. X-ray crystallographic analysis of Apomab-DR5 complex revealed an interaction epitope that partially overlaps with TRAIL binding regions. Apomab-DR5 interface is centered in a nearly continuous region of DR5, comprising residues 62–90, with additional contributions from residues 99–105, in which residues Arg^65^, Ser^68^ and Lys^102^ in DR5 seem to be the key sites. In contrast to Apomab, another phage-derived DR5 antibody (BDF1), which has little or no agonistic activity, interacts TRAIL with different epitope [35].

Computational approaches can provide relevant information to predict binding activity and key functional domains between antigen and antibody complex. It enables researchers to study interactions in atomic detail, and find out how a specific mutation affects its function. Computer-guided models combined with biological experiments could promote the process of identifying the key epitope for certain antigen in binding with its functional antibody. In this study, a novel anti-DR5 monoclonal antibody (mAb, IgG isotype), LaDR5, was screened from DR5-immunized mice, which was more suitable for genetic engineering contrasting to previous mAb (IgM isotype) [36]. The potential anti-tumor activity of LaDR5 without cross-linking was characterized *in vitro* using Jurkat cells. With the major advances in computer processing, the 3-D structure of LaDR5 Fv fragment was simulated based on homology modeling method, and the antigen-antibody (i.e. DR5-LaDR5) complex model were generated using molecular docking method. As the key domain in DR5 being predicted, three mutants of DR5, DR5M1 (residues 35–36 were replaced by alanine), DR5M2 (residues 59, 62, 67–68 were replaced by alanine) and DR5M3 (residues 96, 98, 101 and 104 were replaced by alanine) were designed and prepared by affinity chromatograph, with which the differences of the functional epitope between TRAIL and LaDR5 were investigated.

## Results

### Immunization, screening, purification and antigen binding of LaDR5

Immunization, screening and purification of LaDR5 were carried out using standard protocols described above. Some positive monoclonal antibody clones against DR5 were screened out by enzyme-linked immunosorbent assay (ELISA), in which LaDR5, an IgG1 subtype antibody identified by double immunodiffusion method, was screened out. Hybridoma cells were injected into the peritoneal cavity of BALB/c mice. After 14 days ascites were collected and purified LaDR5 was prepared by a column of protein A-Sepharose 4B.

**Figure 1:**
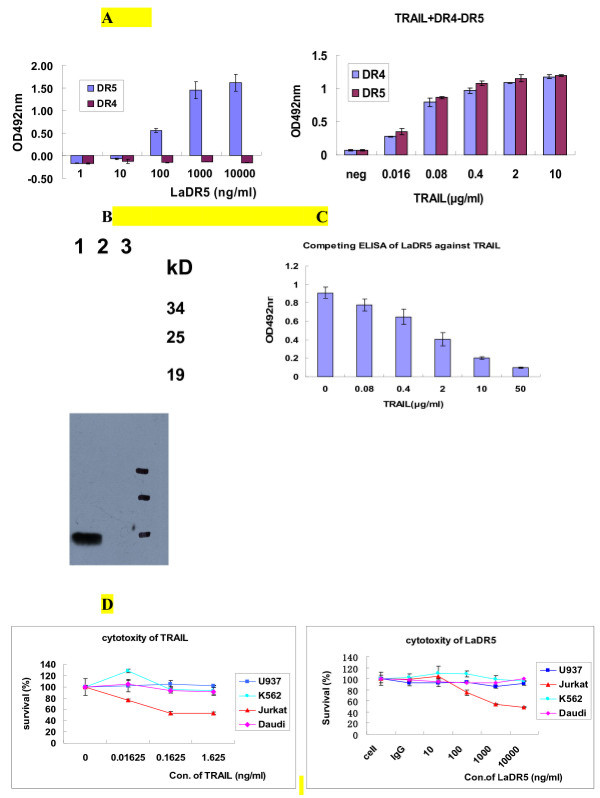
**Antigen binding specificity (A, B&C) and cytotoxic activity of LaDR5 (D).A**: 2 μg/mL DR4 or DR5 was coated and incubated with diluted LaDR5 or TRAIL, by which it was indicated that LaDR5 could bind soluble DR5 specifically other than DR4, while TRAIL could bind both of them in a dose-dependent manner; **B**: Western blot analysis of LaDR5 to bind antigen. 1: DR5; 2: DR4; 3: Protein Marker. Only in lane 1, a specific line with the molecular weight of about 19kD could be seen, indicating that LaDR5 could bind DR5 specifically; **C**: Epitope overlapping of LaDR5 and TRAIL to bind DR5 by competing ELISA. Diluted TRAIL was mixed with LaDR5 previously and the mixtures were added to antigen-coated plate. TRAIL could obviously influence the binding of DR5 and LaDR5 in a dose-dependent manner; **D**: cytotoxic activity of TRAIL or LaDR5 in different tumor cell lines by MTT assay, in which Jurkat seemed to be the most sensitive cell line to LaDR5 as well as TRAIL. Cells were cultured in 96-well flat-bottom plate in triplicates and treated with the indicated concentration of purified LaDR5 or TRAIL. The experimental error is the SD from three independent experiments.

As shown in Figure 1A, when 5 μg/mL DR4 or DR5 was coated, LaDR5 could specifically bind DR5 in a dose-dependent manner rather than DR4. Meanwhile, TRAIL could bind either DR4 or DR5. Furthermore, western blot analysis further displayed its capacity of LaDR5 to bind DR5 with the predicted molecular weight of about 19kD (Figure 1B), indicating that LaDR5 couldn’t cross-react with DR4. Besides, TRAIL could compete with LaDR5 to bind DR5 in a dose-dependent manner, which suggested the epitope overlapping of TRAIL and LaDR5 (Figure 1C).

To identify whether LaDR5 could bind membrane DR5, DR5-low expression cell line K562 and high-expression cell lines Jurkat, U937 and Daudi were used. As shown in Table 1, 10 μg/mL LaDR5 could bind Jurkat (57.05 %), U937 (86.07), and Daudi (63.78 %) cells, respectively, which were approximately consistent with the positive controls (standard anti-DR5 antibody treated samples). LaDR5 seemed not to bind K562 cells, with only 0.4 % positive cells.

**Table 1:** Flow cytometry analysis of the positive cells treated with LaDR5 (10 μg/mL) in different tumor cell lines

**Cell line**	**LaDR5 (%)**	**standard DR5-mAb (%)**
Jurkat	57.05	79.12
K562	0.4	10.92
Daudi	63.78	95.45
U937	86.07	98.18

LaDR5 could bind the membrane DR5 on Jurkat (the percentage of positive cells was 57.05 %), Daudi (63.78 %) and U937 (86.07 %), which was essentially consistent with the standard anti-DR5 antibody treated samples (79.12 % in Jurkat, 95.45 % in Daudi and 98.18 % in U937). In K562 cells, the percentage of positive cells in standard anti-DR5 antibody treated sample was 10.92 % contrasting to 0.4 % in LaDR5 incubated sample.

### Cytotoxic and apoptotic activity of LaDR5

To test the cytotoxic activity of LaDR5 *in vitro*, Jurkat, U937, K562 and Daudi cell lines were cultured with diluted LaDR5. After 24 hours, cell viability was determined by MTT method. As shown in Figure 1D, only Jurkat cells died obviously in a dose-dependent manner (the survival ratio of Jurkat cells could decrease to 46 % when incubated with 10 μg/mL LaDR5, and the IC50 value was about 1 μg/mL). Meanwhile, although Daudi was also membrane-DR5 positive cell line, Jurkat was more sensitive.

**Figure 2:**
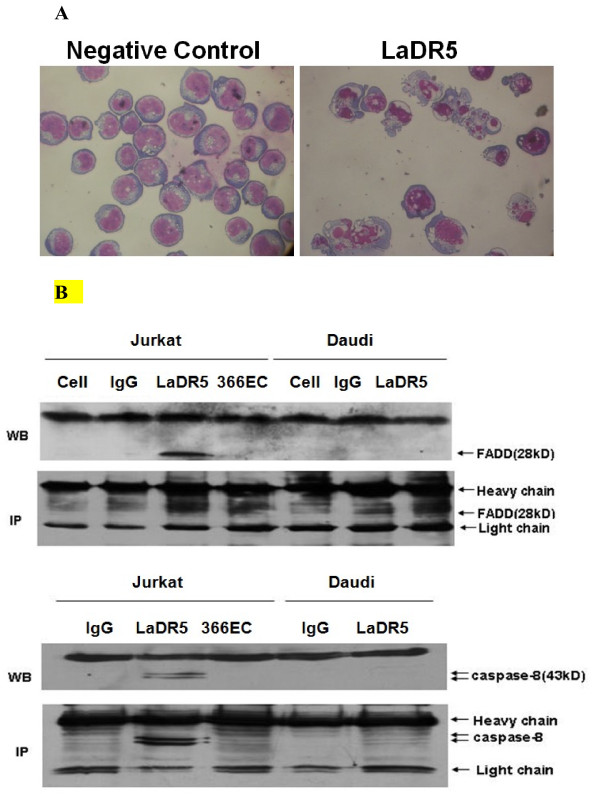
**Apoptotic activity of LaDR5 in Jurkat cells by DISC formation and downstream caspase-8 activation.A**: Giemsa’s staining observation. Jurkat cells were incubated with 10 μg/ml of LaDR5 and sprayed on glass slides. After being fixed, slides were stained with Giemsa’s staining solution for 15 minutes. The samples were examined in light microscopy at 40× magnification, which showed that Jurkat cells displayed typical features of apoptosis. **B**: LaDR5 induces DR5 clustering and DISC activation only in Jurkat cells. Daudi and Jurkat Cells were treated with 10 μg/ml LaDR5, and cell lysates were analyzed first directly by western blot and then immunoprecipitation for FADD/caspase-8 activation, which indicating that only LaDR5 could induce DR5 crosslinking, DISC formation and downstream caspase-8 signals in Jurkat cells. Normal cell, isotype IgG treated cells and DR5-binding Antibody 366EC (with no apoptosis-inducing activity) were set as negative controls.

Giemsa’s staining (Figure 2A) showed that Jurkat cells acquired typical features of apoptosis after being incubated with 10 μg/ml of LaDR5, including cell shrinkage, membrane blebbing and nuclear pyknosis.

Then we analyzed the apoptotic-inducing ability of LaDR5 to form a DR5-associated DISC in cell lysates, indicating that binding of LaDR5 to DR5 results in receptor oligomerization and initiation of apoptosis through the recruitment of FADD; meanwhile, only in Jurkat cells caspase-8 could be detected within 60 minutes (Figure 2B), indicating that LaDR5 was an effective agonist to trigger apoptotic response in Jurkat cells through FADD-caspase-8 pathway, while in LaDR5 insensitive cell line Daudi, the pathway was not triggered. Normal cells, isotype IgG treated cells and DR5-binding Antibody 366EC (with no apoptosis-inducing activity) were set as negative controls.

### The 3-D modeling structures of LaDR5 Fv fragment and DR5-LaDR5 complex

**Figure 3:**
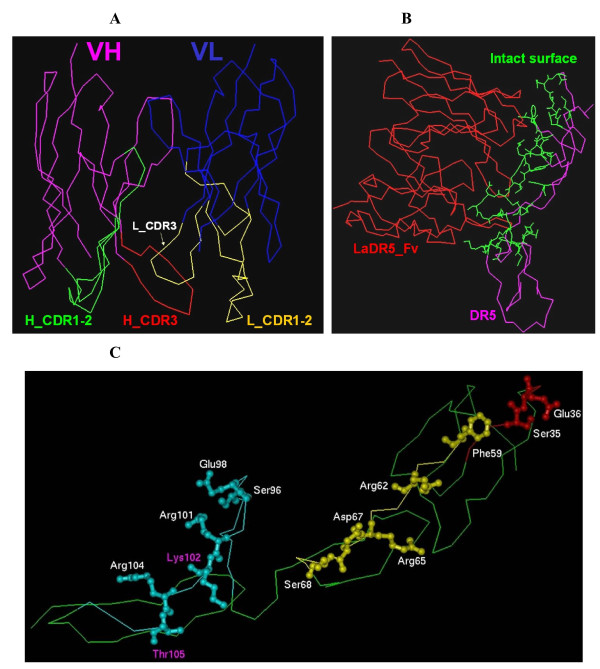
**Computer-aided homology analysis of LaDR5.A**: Homology modeling of LaDR5 Fv, designating V_H_ (pink) and V_L_ fragments (blue). Then CDR domains of heavy and light chains were marked with green (H_CDR1-2), yellow (L_CDR1-2), red (H_CDR3) and white (L_CDR3), respectively; **B**: theoretical structure of LaDR5 (red)-DR5 (pink) complex. The green-line area was the intact suface of the two; **C**: The intact suface between DR5-LaDR5, indicating the key residues of DR5 containing Ser^35^, Glu^36^, Phe^59^, Arg^62^, Arg^65^, Asp^67^, Ser^68^, Ser^96^, Glu^98^, Arg^101^, Lys^102^, Arg^104^ and Thr^105^.

The V_H_ and V_L_ sequences of LaDR5 were determined by traditional isopropyl alcohol-chloroform extraction, RT-PCR, subcloning and sequenced as described above. Then 3-D modeling structures of LaDR5 V_H_ and V_L_ were obtained based on computer-guided homology modeling approach. Using Profile_3D program, the 3-D optimized structures of LaDR5 V_H_ and V_L_ were analyzed. The results showed that more than 94 % of the residues were in the most favored regions. To obtain the structure of LaDR5 Fv fragment, the crystal structure of the esterolytic and amidolytic 43 C9 antibody was selected as template. Consequently, the framework of 1H3P V_L_ and 1A1R V_H_ was superimposed on the corresponding 43 C9 V_L_ and V_H_ framework, using a rigid-body superimposition program [37]. A composite Fv domain was thereby created with V_L_ of 1H3P and V_H_ of 1A1R in which most of the ‘key’ residues and their interactions in the V_L_-V_H_ interface were conserved. Using Homology and docking methods (Molecular Simulations, San Diego, CA), the 3-D structure of LaDR5 Fv fragment was constructed and optimized as shown in Figure 3A.

To probe the mechanism of specific binding and investigate which domain was the most important epitope, the 3-D complex structure of LaDR5 Fv fragment and DR5 was constructed using Docking method and optimized with molecular mechanism and dynamics methods. The stable complex conformation was shown in Figure 3B, and the intact surface between DR5-LaDR5 was shown in Figure 3C, indicating the key residues of DR5: Ser^35^, Glu^36^, Phe^59^, Arg^62^, Arg^65^, Asp^67^, Ser^68^, Ser^96^, Glu^98^, Arg^101^, Lys^102^, Arg^104^ and Thr^105^.

### Epitope Determination of LaDR5

**Figure 4:**
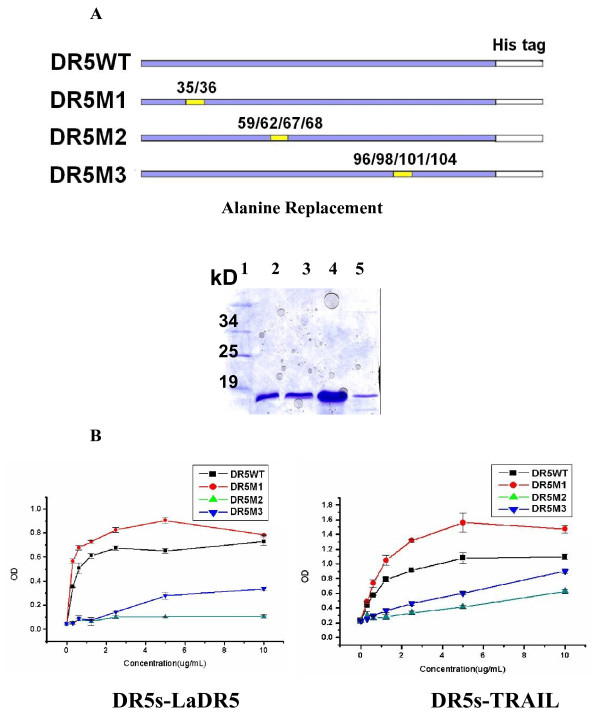
**Epitope determination of LaDR5 or TRAIL.A**: design and purification of wild type DR5 (DR5WT) and its mutants (DR5M1, DR5M2 and DR5M3) in prokaryotic system, indicating the mutable sites above. SDS-PAGE analysis showed satisfactory purification of DR5s. 1: Protein marker; 2: DR5WT; 3: DR5M1; 4: DR5M2; 5: DR5M3. **B**: Epitope identification of LaDR5 by ELISA. 5 μg/mL DR5WT or its mutants was coated overnight and incubated with diluted TRAIL or LaDR5. It was showed that mutation of residues 59/62/67/68 or 96/98/101/104 in DR5 attenuated the DR5 binding capacity of LaDR5 or TRAIL, suggesting that they were key residues of DR5 identified by both TRAIL and LaDR5; besides, residues 59/62/67/68 seemed more important, which were consistent with theoretical results predicted by computer-aided analysis. The experimental error is the SD from three independent experiments.

To verify the epitope of DR5 identified by LaDR5, the expression vectors of DR5WT and its alanine-replaced mutants (DR5M1, DR5M2 and DR5M3) were constructed. Proteins were obtained from prokaryotic system *E.coli* BL21. SDS-PAGE analysis displayed a single band of each purified protein with the molecular weight of about 19kD (Figure 4A). ELISA results were showed that mutation of residues 59/62/67/68 (DR5M2) or 96/98/101/104 (DR5M3) in DR5 attenuated the DR5 binding capacity of TRAIL or LaDR5 (Figure 4B), suggesting those sites key residues of DR5 identified by both TRAIL and LaDR5. The TRAIL or LaDR5 binding capacity order of DR5WT and DR5 mutants seemed to be DR5M1 > DR5WT > DR5M3 > DR5M2, indicating the importance of residues 59/62/67/68 and 96/98/101/104. Furthermore, for DR5M2 almost lose the ability to bind LaDR5 or TRAIL, residues 59/62/67/68 seemed more important, especially in LaDR5 reaction, which were consistent with theoretical results predicted by computer-aided analysis. Meanwhile, Relative affinity constant of LaDR5 to bind DR5 mutants were determined by ELISA. As shown in Table 2, the order of affinity were DR5M1 > DR5WT > DR5M3 > DR5M2, which were consistent with binding assays. To our surprise, the calculated relative affinity constant of LaDR5 to bind DR5M2 were minus, with the possible reason that the constant approached or quite near zero, which indicated quite low affinity of DR5M2 to bind LaDR5.

**Table 2:** Relative affinity constant (Kd) of LaDR5 to bind DR5s using ELISA method

**Antigen**	**Relative Affinity Constant of LaDR5 (nmol/L)**
DR5WT	454.6
DR5M1	392.7
DR5M2	- *
DR5M3	7043.6

* Relative affinity constant was minus, with the possible reason that the constant approached or quite near zero, indicating low affinity of DR5M2 to bind LaDR5.

The order of affinity were DR5M1 > DR5WT > DR5M3 > DR5M2, which were consistent with ELISA results.

**Figure 5:**
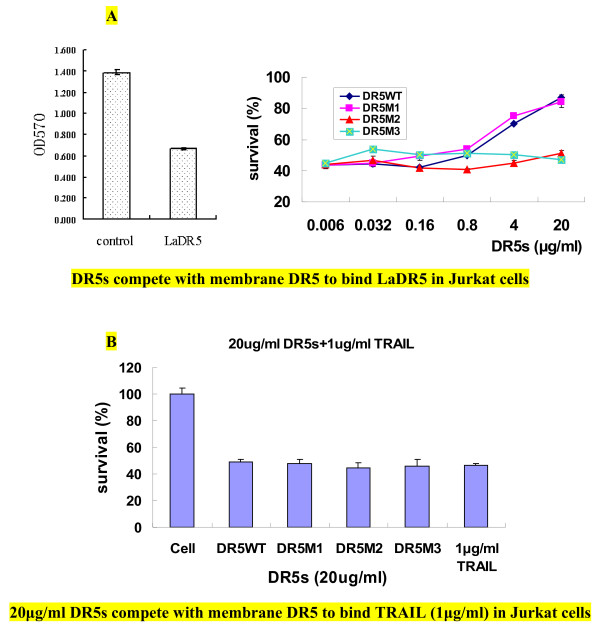
**Inhibitory activity of DR5s to the cytotoxicity of LaDR5 or TRAIL in Jurkat cells.** 0.64 μg/mL LaDR5 or 0.16 μg/mL TRAIL was chosen according to the standard curve in Figure 1D to induce apoptosis of 50 % cells. **A**: Competing activity of DR5s against membrane DR5. The left panel displayed the OD value of LaDR5 treated sample contrasting to normal cells (control); the right panel indicated the competition of DR5s against membrane DR5 to rescue cells from apoptosis. DR5WT and its mutants DR5M1 could interfere the cytotoxicity of LaDR5 in Jurkat cells, while DR5M2 and DR5M3 couldn’t, indicating that DR5M2 or DR5M3 lacked the capacity to bind LaDR5, which was also consistent with ELISA results shown in Figure 4; **B**: None of DR5WT or its mutants could inhibit the cytotoxicity of TRAIL in Jurkat cells. The experimental error is the SD from three independent experiments.

Besides, the inhibitory activity of DR5WT or its mutants to the cytotoxicity of LaDR5 or TRAIL in Jurkat cells was also carried out. As shown in Figure 5A, DR5WT and DR5M1 could obviously interfere the cytotoxicity of LaDR5 in Jurkat cells, while DR5M2 and DR5M3 couldn’t, indicating that only DR5M1 and DR5WT could compete with membrane DR5 to neutralize LaDR5 on Jurkat cells,therefore residues 59/62/67/68 (DR5M2) or 96/98/101/104 (DR5M3) were predominant sites in LaDR5 binding, which were consistent with ELISA results shown in Figure 4B. However, to our surprise, none of DR5WT or its mutants could inhibit the cytotoxicity of TRAIL in Jurkat cells (Figure 5B).

## Discussion

DR4 and DR5 are capable of transducing an apoptosis signal. Activation of DR4 or/and DR5 in various cancer cells triggers programmed cell death through the extrinsic pathway. Agents that activate DR4 or DR5, including TRAIL, have attracted substantial attention and investment as potential anti-cancer therapies. For TRAIL has no cytotoxicity in many normal cell types except for human liver cells [30], other agonistic reagents against the pro-apoptotic molecules DR4 or DR5, such as anti-DR5 mAbs, became an attractive anti-cancer strategy because of their potential for inducing tumor-specific cell death. Human or humanized anti-DR5 mAbs Lexatumumab (HGS-ETR2), Apomab, Conatumumab (AMG 655) and Tigatuzumab (CS1008) have been identified functional as a single agent or in combination with chemotherapy or other agents. Epitope of Apomab resides in residues 62–90 and residues 99–105 of DR5, which partially overlaps with that of TRAIL binding sites. DR5 residues Arg65, Ser68 and Lys102 seem to be most functional residues, while the nonfunctional anti-DR5 antibody BDF1 interacts different epitope from Apomab [35].

Computational approaches and protein structural analysis can provide relevant information about the functional roles of the epitope residues. Fast protocols using knowledge-based approaches and distance geometry analysis for predicting binding activity and key functional domains have been established. The physical interactions between antigen and antibody in their complex structures provide crucial insights into antigen function. It is precisely these structures that enable researchers to study interactions in atomic detail, and find out, for example, how a specific mutation in antigen affects its function, or how functional domain formed pharmacophore. Here computer-guided models were combined with biological experiments for the efficient determination and identification of the key domain for antigen (i.e. DR5) in binding with its functional antibody.

Thus, in this study, a functional anti-DR5 mAb, LaDR5, was screened out. The apoptosis-inducing and potential anti-tumor activity of LaDR5 without cross-linking was characterized *in vitro*. As shown in Figure 1, only membrane DR5-positive cell line Jurkat died with LaDR5 obviously in a dose-dependent manner, while DR5-low expression cell line K562, high expression cell line Daudi and U937 were insensitive to LaDR5. Furthermore, Jurkat cells displayed apoptosis feature when LaDR5 was added (Figure 2A). The DR5-associated DISC was investigated by western blot and immunoprecipitation in LaDR5-treated Jurkat or Daudi cell lysates, by which LaDR5 could induce the recruitment of FADD and then caspase-8 activation only in Jurkat cells (Figure 2B), which was consistent with the cytotoxic and apoptotic results. In Daudi cells, FADD-caspase-8 signal pathway was not triggered, the reason might be the failure of surface DR5 crosslinking by LaDR5 to form DISC or the interference of negative regulating molecules against caspase-8 activation (e.g. c-FLIP) [38].

Based on the computational methods and protein structural analysis, the key epitope of LaDR5 was predicted. First, the 3-D modeling structures of LaDR5 V_H_ and V_L_ were obtained, followed by constructing the 3-D structure of LaDR5 Fv fragment (Figure 3A). Then using Docking method, the 3-D complex structure of LaDR5 Fv fragment and DR5 (LaDR5-DR5) was constructed and optimized (Figure 3B); meanwhile, the key residues of DR5 identified by LaDR5 was determined theoretically (Figure 3C), according to which three DR5 mutants were designed and purified to confirm the prediction. The TRAIL or LaDR5 binding capacity order of DR5WT and DR5 mutants seemed to be DR5M1 > DR5WT > DR5M3 > DR5M2, suggesting that residues 59/62/67/68 (DR5M2) and 96/98/101/104 (DR5M3) in DR5 were key residues to bind TRAIL or LaDR5 (Figure 4B and Table 2). Furthermore, residues 59/62/67/68 (DR5M2) were more important in LaDR5 reaction, which were consistent with theoretical results predicted by computer-aided analysis. Furthermore, only DR5M1 as well as DR5WT could obviously interfere the cytotoxicity of LaDR5 in Jurkat cells, indicating residues 59/62/67/68 (DR5M2) or 96/98/101/104 (DR5M3) were predominant functional sites in the cytotoxic activity of LaDR5 (Figure 5A) as well as in the binding of LaDR5. Indeed the design procedure of DR5M1 was similar as that of the other mutants. The domain (i.e. Ser^35^, Glu^36^) of DR5 was the boundary of the binding sites, when the residues mutated in DR5M1 were replaced with alanine, the hydrophilic property of the domain was lost and the hydrophobic area was increased, then the change benefited to the binding affinity.

However, to our surprise, none of DR5s could inhibit the cytotoxicity of TRAIL (Figure 5B), with the possible reasons we conferred could be as follows: firstly, TRAIL identifies wider binding area of DR5 with higher affinity and/or triggers too strong cytotoxic signal to be interfered; secondly, no matter whether TRAIL dissociates or falls off after binding to membrane DR5, when TRAIL starts to bind, the downstream signals are induced immediately resulting in TRAIL-inducing cytotoxicity, although this statement should be supported by further experiments; thirdly, although the binding sites of DR5 identified by TRAIL known from the X-ray crystallographic analysis was overlapped by LaDR5 binding sites, the cytotoxic processes induced by TRAIL should be more than DR5-inducing pathway, for example, TRAIL can trigger cell death through DR4 rather than DR5, therefore in TRAIL-DR4 inducing cytotoxicity, it was difficult to inhibit downstream apoptosis signals by single DR5 analogs, which were more conceivable in our consideration.

## Conclusions

Our work demonstrated the specific epitope located in DR5 that plays a crucial role in antibody binding; furthermore, revealed structural features of LaDR5-DR5 complex may be beneficial to design or screen novel antineoplastic drugs aganist DR5.

## Methods

### Materials

Fluorescein isothiocyanate (FITC) or HRP labeled goat anti-mouse antibody was purchased from Sigma-Aldrich; Mouse anti-caspase-8 monoclonal antibody (mAb) was from BD Bioscience; Mouse anti-DR5 monoclonal antibody was from eBioscience; Rabbit anti-FADD mAb was from Upstate; HRP conjugated anti-6 × His antibody (anti-His_HRP) was purchased from Abcam; Human T lymphocyte cell line Jurkat (ATCC No. TIB-152), chronic myelogenous leukemia K562 (ATCC No. CCL-243), human B lymphoblast Daudi (ATCC No. CCL-213), histiocytic lymphoma U937 (ATCC No. CRL-1593.2) and *E.coli* BL21 were stored in our laboratory.

### Production and purification of mAb

Immunization and production of mAbs were carried out using standard protocols. In short, five 4-week-old female BALB/c mice were immunized subcutaneously with 100 μg of purified DR5 in complete Freund’s adjuvant per animal. Then animals were boosted twice at 4-week intervals with 100 μg of antigen in incomplete Freund’s adjuvant. Three days after the final injection, one mouse was sacrificed and its splenocytes were fused with NS-1 at a 6:1 ratio to a final concentration of 2 × 10^6^ cell/mL, and 200μL cells was plated in each well of five 96-well plates. Hybrids were selected in RPMI1640 Medium supplemented with 20% fetal calf serum and 5 × 10^-3^ M hypoxanthine, 2 × 10^-5^ M aminopterin, and 8 × 10^-4^ M thymidine (HAT). After about 10 days, cell clones secreting antibodies against E34 were screened by ELISA, and the positive clones were selected and subcloned to establish stable cell lines.

5 × 10^6^ hybridoma cells were injected into peritoneal cavity of BALB/c mice treated with paraffin in advance. After about twelve days, ascites were withdrawn and centrifuged at 1500 rpm for 5 minutes at 4°C. The supernatant was then applied to a column of protein A-Sepharose 4B. Specific-binding antibody was eluted with pH 4.0 citric acid buffer and dialyzed against PBS overnight.

We observed animal ethics during the research by complying with 3R principles (Replacement, Reduction and Refinement), such as usage of SPF-level mice, mice anesthetization, less animal number and mercy killing, etc. And the ethical approval granted for the study was given by the **Ethics Committee of Basic Medical Sciences (Immunology)**.

### ELISA

ELISA plates (Nunc, Roskilde, Denmark) were coated with 5 μg/mL of DR4, DR5WT or its mutants at 4°C overnight and blocked with 3% BSA. 100μL diluted LaDR5 or TRAIL was added, followed by 100μL HRP_conjugated anti-mouse polyclonal antibody for 45 minutes at room temperature (RT for short, the same below). The peroxidase reaction was developed with color development solution. The light absorbance was measured with an ELISA reader.

### Relative affinity constant of LaDR5 and TRAIL to bind DR5s determined by ELISA [39]

To evaluate the affinity constant of LaDR5 to bind DR5s, a modified ELISA method was used. In brief, according to the binding curve, concentration of LaDR5 in saturation point (such as 0.6 μg/mL LaDR5 to bind DR5) was chosen to carry out the detection of affinity constant. Then LaDR5 was mixed with diluted DR5s and incubated at 4°C overnight. The complexes were added to DR5s-coated plate. After three washes, GAM_HRP was added. The following formula was used to calculate the affinity constant:

(1) $$\text{Kd}={\text{a}}_{0}\times \left[\frac{\text{A}}{\left({\text{A}}_{0}-\text{A}\right)}-1\right]\text{.}$$

Here, in certain condition of diluted antigen, a_0_ means the antigen concentration; A is the absorbance value in this specific condition; A_0_ means the absorbance value of positive control with no antigen in the pre-incubated complex. The mean value of all Kd values was the final affinity constant.

### Western blot

DR4 or DR5 were resolved by 12% SDS-PAGE and then transferred to a nitrocellulose membrane. After blocking with 5 % milk in PBS at RT for 1 hour, the blots were probed with 5 μg/mL LaDR5 followed by horseradish peroxidase (HRP)-conjugated goat anti-mouse polyclonal antibody and developed by chemiluminescence.

### Flow cytometry analysis

Cells (5 × 10^5^) were collected by centrifugation and incubated with 5 μg/ml of LaDR5 or standard anti-DR5 antibody. Mouse IgG1 was set as negative control. Then cells were incubated with 100μL FITC-conjugated goat anti-mouse IgG1 for 30 min at 4°C with gentle shaking. Samples were analyzed on a FACSCalibur (Becton-Dickinson) using the software program CellQuest.

### Cytotoxic activity

A total of 1 × 10^5^ cells per well were cultured in flat-bottom 96-well plate in triplicates with the indicated concentration of purified antibody LaDR5 or TRAIL. 10μL per well MTT (10 mg/ml) was added and incubated for additional 4 hours at 37°C. Then 100μL 10% SDS containing 0.01 mol/L HCl was added and the plate was incubated for another 12 hours for light absorbance measurement at 570 nm with an ELISA reader.

For competing experiments, the concentration of LaDR5 or TRAIL was chosen with the OD value of about 50 % of saturated value, because it was sensitive to concentration change. Diluted DR5s were added at the same time to compete with membrane DR5 in binding LaDR5 or TRAIL, which would rescue cells from LaDR5-triggering apoptosis.

### Giemsa’s staining

Jurkat cells were incubated with 10 μg/ml of LaDR5 for 30 min at 37°C. Cells were collected by centrifugation at 4°C for 5 min at 1,000 rpm, then after washing, cells were sprayed on glass slides with a Cytospin (Shandon, Astmoor, England), air dried and fixed with methanol for 1 min, stained with 10% Giemsa’s staining solution for 15 minutes, washed with distilled water, and finally air dried. The samples were examined in a light microscopy at 40× magnification.

### Immunoprecipitation (IP)

Jurkat or Daudi cells (1 × 10^7^ per sample) were treated with LaDR5 (10 mg/ml) for 1 hours at 37°C. Then cells were collected by centrifugation, lysed in IP buffer containing 20 mM Tris–HCl, 150 mM NaCl, 10 % glycerol, 2 mM EDTA, 0.57 mM PMSF, 1 % NP-40, pH8.0 and immunoprecipitated for 4 hours on ice. Then 15μL protein A beads (Sigma) per sample was added to the supernatant and after a hour’s washing, the beads were analyzed by western blot. Normal cell, isotype IgG treated cells and DR5-binding Antibody 366EC (with no apoptosis-inducing activity) were set as negative controls.

### Gene cloning and sequencing of LaDR5

The V_H_ and V_L_ sequences of LaDR5 were determined by traditional method. In short, hybridoma cells were cultivated, collected and lyzed in TRIzol (Invitrogen). Then total RNA of LaDR5 were obtained using isopropyl alcohol-chloroform extraction. Then using reverse transcription and PCR with specific mouse antibody primers, the VH and VL region were subcloned into pGEM-Teasy vector (Promega) and sequenced.

### Computer-guided molecular homology modeling

cDNAs encoding the variable domains of the light and heavy chains (V_L_ and V_H_) of LaDR5 were derived from hybridoma cell by RT-PCR using consensus primers. The amino-acid residue sequences of the V_L_ and V_H_ of LaDR5 were compared with the primary sequences of all immunoglobulins deposited in the Protein Data Bank [40] using BLAST program (http://www.ncbi.nlm.nih.gov/BLAST/) [41]. The CDR (Complementary Determinant Region) definition of LaDR5 variable domains was studied using Kabat method (http://immuno.bme.nwu.edu/) [42].

The best match for the V_H_ domain of LaDR5 was the Fab-peptide complex structure (PDB code: 2hrp) [43], sharing 81% of sequence identity with the template, whereas the most homologous V_L_ of LaDR5 was the crystal structure of an anti-HCG Fab (PDB code: 1sbs) [44], sharing 84% of sequence identity with the template. The structures were used as templates for computer-guided homology modeling of the 3-D theoretical structure of LaDR5 Fv fragment with HOMOLOGY program. The orientations of the V_H_ and V_L_ domains were generated by superimposition choosing the crystal structure of IgG1 Fab fragment (PDB code: 1igc) [45] as a scaffold template.

To optimize the steric clashes, the 3-D modeling structure of LaDR5 Fv fragment was subjected to energy minimization with 5000 steps of steepest descent followed by 10000 steps of conjugate gradient until the convergence criterion of 0.05 kCal/mol·Å was obtained under Charmm forcefield. Structural validation of LaDR5 Fv fragment was evaluated using Profile_3D program.

### Molecular docking

Based on the crystal structure of DR5 from PDB database (PDB code: 1d0g) [46], the theoretical structure of DR5 was optimized (5000 steps of steepest descent followed by 10000 steps of conjugate gradient) under Charmm forcefield. The 3-D complex structure of DR5 and LaDR5 was constructed using molecular docking method, where the CDR loops of LaDR5 were defined as the potential binding sites. DR5 and the potential binding sites were set to be flexible during the docking simulation and the modeling complex structure was energetically evaluated based on van der Waals and hydrogen bond interaction energy.

To avoid the conformation of the interaction domains trapped in a local potential energy minimum, residues at the base of the interaction domains were held fixed while the remainder of the interaction domains was subjected to simulated heating and molecular dynamics at elevated temperatures followed by slow cooling to a low energy conformation. The interaction domain residues were initially assigned a temperature of 300 K and slowly heated to 500 K in increments of 25 K, with 50 dynamics steps at each temperature using a time step of 1 fs. The structure was similarly heated to 1000, 2000, 3000 and 4000 K. At each temperature the interaction domains were subjected to a 100 ps dynamics run followed by slowing to 300 K, and two series of minimizations, first for 500 steps, then 3000 steps.

Based on the 3-D optimized complex structure of DR5 and LaDR5, the binding sites between DR5 and LaDR5 were predicted and a series of DR5 mutants were designed. The mutated sites in DR5M2 and DR5M3 were located at the coiled region of DR5, thus, the conformations of the mutants DR5M2 and DR5M3 remained the main orientation of the wild type of DR5 and did not change. The effect of DR5 did not affect.

### Prokaryotic expression and purification of DR5WT and three mutants

Extracellular fragment of DR5 (DR5WT) and its three mutants (DR5M1, DR5M2 and DR5M3) were prepared as described [47]. The expression plasmids were constructed using overlap PCR, enzymatic digestion and ligation. After sequencing, plasmids were transformed into *E.coli* BL21, respectively, and induced by 0.1 mM isopropyl-β-D-thiogalactopyranoside (IPTG) overnight at 20°C. Cells were collected by centrifugation, resuspended and lysed by sonication at 4°C. After centrifugation, supernatant was immediately loaded onto a Ni^2+^ nitrilotriacetic acid Sepharose affinity column. After being washed with 10 volumes of a binding buffer, the column was loaded with diluted imidazole (10 mM, 30 mM, 50 mM, 200 mM and 500 mM) in 20 mM PB, pH 8.0. The fusion proteins were collected and desalted using MILLPORE column (MWCO, 10000). The concentration of purified protein was calculated by ultraviolet spectrophotometer.

## Competing interests

The authors declare that they have no competing interests.

## Authors' contributions

CQ screened the antibody LaDR5 and drafted the manuscript, MH expressed and purified the mutants of DR5 and identified the epitope of LaDR5, LG did in vitro experiments (cytotoxic and apoptotic activity), ML constructed the plasmids, ZL did in vitro experiments (antigen binding) and revised the manuscript, JG cultivated cell lines, XLL prepared the antibody, XYL analyzed the data of flow cytometry, YL conceived of the study and participated in its design, YM participated in its design and coordination, JF did computer-aided analysis and design, BS helped draft the manuscript. All authors read and approved the final manuscript.
